# Clinical significance of miR-3178 in non-small cell lung cancer patients

**DOI:** 10.5937/jomb0-57060

**Published:** 2026-06-16

**Authors:** Deng Huang, Yingfang Ma, Haizhen Yu, Shizhen Li, Ling Zhou

**Affiliations:** 1 Yangxin People's Hospital, Department of Respiratory and Critical Care Medicine, Huangshi City, China; 2 Heilongjiang Provincial Daqing Hospital of Traditional Chinese Medicine, Interventional Radiology Department, Heilongjiang, China; 3 Zhucheng People's Hospital, Department of Laboratory, Zhucheng, China; 4 Wuhan Pu Ren Hospital, Pulmonary and Critical Care Medicine, Wuhan, China

**Keywords:** non-small cell lung cancer, miR-3178, MIB2, nemikrocelularni karcinom pluća, miR-3178, MIB2

## Abstract

**Background:**

Non-small cell lung cancer (NSCLC) is a malignant tumour with high morbidity and mortality, with a low survival rate and poor prognosis. This study aims to explore the diagnostic and prognostic value of miR-3178 in NSCLC.

**Methods:**

The study included 118 NSCLC patients and 97 healthy subjects. RT-qPCR measured miR-3178 and MIB2 in serum or cell lines. c 2 tests linked miR-3178 to clinico-pathological features. ROC, Kaplan-Meier, and Cox analyses evaluated the diagnostic and prognostic significance of miR-3178 and MIB2.

**Results:**

Compared to the control group, the expression level of miR-3178 was significantly reduced, while MIB2 expression was notably elevated in NSCLC patients. The expression of miR-3178 is significantly correlated with the TNM stage and LNM in NSCLC patients. NSCLC patients with higher miR-3178 expression levels exhibit significantly higher 5-year overall survival rates. Furthermore, TNM stage, miR-3178 expression, and MIB2 expression are independent risk factors that influence the prognosis of NSCLC. MIB2 serves as a target gene of miR-3178, and its expression levels display a significant negative correlation. When diagnosing NSCLC solely based on miR-3178, the AUC is 0.838, the diagnostic sensitivity and specificity were 83.1% and 72.2% , respectively; for MIB2 alone, the AUC is 0.818, the sensitivity and specificity were 76.3% and 72.2% ; however, when miR-3178 and MIB2 are combined for diagnosis, the AUC increases to 0.903, the sensitivity and specificity were 84.8% and 83.5%.

**Conclusions:**

The downregulation of miR-3178 and upregulation of MIB2 are associated with the progression and deterioration of NSCLC patients, indicating poor prognosis. miR-3178 targets MIB2, and their combined diagnosis can enhance the diagnostic value for NSCLC, potentially reducing the risk of adverse outcomes.

## Abstract (sr)

**Uvod:**

Nemikrocelularni karcinom pluća (NSCLC) je maligni tumor sa visokom stopom oboljevanja i smrtnosti, niskom stopom preživljavanja i lošom prognozom. Cilj ove studije je da istraži dijagnostičku i prognostičku vrednost miR-3178 kod pacijenata sa NSCLC.

**Metode:**

Studija je obuhvatila 118 pacijenata sa NSCLC i 97 zdravih ispitanika. RT-qPCR metodom mereni su nivoi miR-3178 i MIB2 u serumu ili ćelijskim linijama. c 2 testom analizirana povezanost miR-3178 sa kliničko-patološkim karakteristikama. ROC analiza, Kaplan-Majer i Cox analiza su korišćene za procenu dijagnostičke i prognostičke vrednosti miR-3178 i MIB2.

**Rezultati:**

U poređenju sa kontrolnom grupom, kod pacijenata sa NSCLC nivo miR-3178 je bio značajno smanjen, dok je ekspresija MIB2 bila značajno povišena. Ekspresija miR-3178 bila je značajno povezana sa TNM stadijumom i LNM kod pacijenata sa NSCLC. Pacijenti sa višim nivoom miR-3178 su pokazali značajno veću petogodišnju stopu ukupnog preživljavanja. Pored toga, TNM stadijum, ekspresija miR-3178 i ekspresija MIB2 predstavljaju nezavisne faktore rizika koji utiču na prognozu NSCLC. MIB2 je identifikovan kao ciljni GEN miR-3178, a njihova ekspresija pokazuje značajnu negativnu korelaciju. Pri dijagnostikovanju NSCLC samo na osnovu miR-3178, AUC je 0,838, dijagnostička osetljivost i specifičnost su iznosile 83,1% i 72,2% ; za MIB2 pojedinačno, AUC je 0,818, a osetljivost i specifičnost 76,3% i 72,2% ; međutim, kombinacija miR-3178 i MIB2 za dijagnozu povećava AUC na 0,903, a osetljivost i specifičnost iznose 84,8% i 83,5%.

**Zaključak:**

Smanjena ekspresija miR-3178 i povećana ekspresija MIB2 su povezane sa progresijom i pogoršanjem stanja kod pacijenata sa NSCLC, što ukazuje na lošu prognozu. miR-3178 cilja MIB2, a njihova kombinovana dijagnostika može poboljšati dijagnostičku vrednost za NSCLC i potencijalno smanjiti rizik od nepovoljnih ishoda.

## Introduction

Lung cancer ranks as the second most common malignancy globally and constitutes a significant contributor to cancer-related fatalities in China [1][2]. As one of the most common types of lung cancer, nonsmall cell lung cancer (NSCLC) accounts for 85% of all lung cancer cases. It is a heterogeneous disease that encompasses different histological subtypes, primarily adenocarcinoma, squamous cell carcinoma, and large cell carcinoma [3][4]. NSCLC has no obvious early symptoms, leading to late diagnosis and poor prognosis [5]. Despite rapid advances in precision oncology and some progress made in the treatment and prognosis of NSCLC patients, it remains a clinically challenging disease, with 5-year survival rates of 15% and 83% for advanced and stage I NSCLC patients, respectively [6]. Therefore, it is of great importance to identify new biomarkers that aid in the diagnosis, prognosis, and treatment of NSCLC.

Currently, molecular screening is a research hotspot in early screening for NSCLC, with circulating microRNAs (miRNAs) demonstrating significant effectiveness in early cancer screening [7][8]. miRNAs are widely present in eukaryotic cells and stably exist in human circulation. Compared to other markers, miRNAs possess the characteristics of stability, repeatability, and consistency, and have gradually been used for early cancer screening in recent years [9]. It is reported that miRNAs in NSCLC patients have oncogenic or tumour suppressor effects through their target genes. For example, the let-7 family, miR-34, miR-486, and miR-200 act as tumour suppressors, while miR-196b, miR-21, and miR-224 act as oncogenes [10]. miR-1231 plays a suppressive role in prostate cancer and is accompanied by significant downregulation [11]. miR-532 is regarded as a prognostic biomarker, and its upregulation promotes migration and invasion of gastric cancer [12]. According to current research, abnormally expressed miRNAs in serum may serve as diagnostic markers for NSCLC [13].

miR-3178 is abnormally expressed in many tumours, but there is relatively little research on miR-3178 in lung cancer. Some studies have found that miR-3178 is down-regulated in lung cancer [14][15]. However, the involvement and regulatory mechanisms of miR-3178 in NSCLC pathogenesis remain largely unexplored. MIB2 is a RING-type ubiquitin ligase that regulates the Notch signalling pathway through ubiquitination of Notch ligands and is also involved in the regulation of NF-B signalling pathways and type I interferon responses [16]. Current research indicates that MIB2 plays an essential role in various malignant tumours, such as cervical cancer, breast cancer, and neuroblastoma [17][18][19]. The role of serum matrix-derived miR-3178 and MIB2 in the early screening and diagnosis of NSCLC warrants further investigation.

In the study, the differential expression of miR-3178 and MIB2 in NSCLC was investigated. The correlation between miR-3178 expression and NSCLC severity was evaluated. The Kaplan-Meier survival curve has been used to assess the predictive value of miR-3178 for the 5-year prognosis of NSCLC patients. Furthermore, the downstream target of miR-3178 was identified through a database, and the interaction between miR-3178 and MIB2 was explored by dual luciferase activity. The combined diagnostic value of miR-3178 and MIB2 for NSCLC was analysed, aiming to provide an effective diagnostic basis for NSCLC in clinical practice.

## Materials and methods

Study subjects: A total of 118 patients admitted to Zhucheng People's Hospital from January 2017 to December 2018 were selected as the experimental group, and 97 healthy individuals who underwent physical examination during the same period were selected as the control group. Inclusion criteria: (1) All patients in the experimental group were confirmed to have NSCLC through cytological or pathological examinations; (2) Aged between 50 and 75 years, regardless of gender; (3) All participants signed an informed consent form; (4) Relevant examinations were complete, and data were available. Exclusion criteria: (1) Patients who had undergone radiotherapy, chemotherapy, or targeted therapy before sample collection for the study indicators; (2) Patients with infection or acute inflammatory diseases; (3) Patients with other malignancies; (4) Patients with haematological or immune system diseases; (5) Pregnant or lactating women. For the healthy control group, the inclusion criteria are as follows: (1) matched with the NSCLC patients in age, gender, BMI, and smoking and drinking history; and (2) confirmed by the medical examination center that the individual's lung function and biochemical indicators are normal, no viral pneumonia, and no history of lung or other malignant diseases. This study has obtained the informed consent of patients and their close relatives, as well as the approval of the hospital ethics committee. All patients were followed up for five years or until their death, and the time of death was recorded through telephone or an outpatient visit.

Sample collection: Blood samples were collected from both groups, centrifuged at 4,000 r/min for 10 minutes, and the supernatants were collected and stored in a -80°C freezer for future testing.

Cell transfection: Three NSCLC cell lines, including A549, H1299, and MES-1, as well as normal human lung epithelial cells BEAS-2B from the Chinese Academy of Sciences Shanghai Cell Bank, were purchased. Cells were cultured in a 37°C humidified incubator with 5% CO_2_ in RPMI1640 medium (Hyclone, Logan, UT, USA) supplemented with 10% fetal bovine serum (FBS, Gibco, Grand Island, NE, USA). In addition, the miR-3178 mimics and their negative controls (NC) were acquired from GenePharma (Shanghai, China) and transfected into A549 or H1299 cells via Lipofectamine 2000 (Invitrogen, USA). The miR-3178 mimics sequence was 5'-GGGGCGCGGCCGGAUCG-3' and the miR-NC sequence was 5'-CCTTGGATGGCCTAGGAGATAG-3'. QRT-PCR verified the transduction efficiency.

Dual-Luciferase reporter assay: The relationship between miR-3178 and MIB2 was evaluated using a dual-luciferase reporter assay. The binding sites of miR-3178 and MIB2 were predicted by TargetScan, and wild-type (WT) and mutant (MT) MIB2 were established. The mutant type of MIB2 was established through point mutations in the binding sites. The reporter plasmid was co-transfected with miR-3178 mimics or negative controls into NSCLC cell lines for 48 hours. The luciferase activity was detected using the dual-luciferase reporting kit (Promega, Shanghai, China) according to the instructions. The activity of Renilla luciferase was considered an internal control for the calculation of relative firefly luciferase activity.

RNA extraction and qRT-PCR: Total RNA was extracted from cells and serum using Trizol reagent (Invitrogen, Carlsbad, CA, USA). cDNA was synthesised by reverse transcription using reverse transcriptase (TOYOBO, Japan). PCR amplification was performed using SYBR premix (TOYOBO, Japan) at 60°C for 10 min, 95°C and 72°C for 30 s, and 95°C for 5 min. This experiment used specific primers of miR-3178 (forward primer 5'-TCG-GCAGGGGGGCG-CGGCC-3' and reverse primer 5'-GCGTGTCGTGGAGTCGGC-3'). Using U6 (GENEWIZ) as an internal control (forward primer 5'-CATCAC-CATCACTCAGG-AGAGTcG-3' and reverse primer 5'-TGACGCTTGCCCACAGCCTT-3'), the expression levels of miR-3178 and MIB2 were calculated by the 2-^ΔΔCt^ method.

Data processing: Data were analysed using SPSS 22.0 software. The results are expressed as mean ±SD, and comparisons between groups were performed using independent sample t-tests. The Bonferroni method was a post hoc test for comparing differences. A chi-square test identified the clinico-pathological characteristics that correlated with miR-3178 expression. Pearson correlation coefficients were used for correlation analysis, and ROC analysis was employed to assess the diagnostic value of miR-3178 and MIB2 in NSCLC. Kaplan-Meier analysis and Cox regression analysis were conducted to determine the prognostic significance of miR-3178. *P*<0.05 was considered statistically significant.

## Results

### General information on healthy controls and NSCLC patients

The study included 60 male and 37 female healthy controls with an average age of 61.58±4.57 years and a BMI of 22.59±1.32 kg/m^2^. The NSCLC patients comprised 71 males and 47 females, with an average age of 61.22±4.72 years and a BMI of 22.87±1.37 kg/m^2^. No significant differences were observed between healthy controls and NSCLC patients in terms of age, gender, BMI, smoking history, or drinking history (*P*>0.05, Table 1).

**Table 1:** Comparison of baseline data between the two groups of people. Note: BMI, body mass index

	Healthy control (n=97)	NSCLC patients (n=118)	*P*-value
Age	61.58±4.57	61.22±4.72	0.576
Gender (male/female)	60/37	71/47	0.801
BMI	22.59±1.32	22.87±1.37	0.134
Smoking history	46	57	0.307
Drinking history	45	63	0.897

### Expression levels of miR-3178 and MIB2

**Figure 1:**
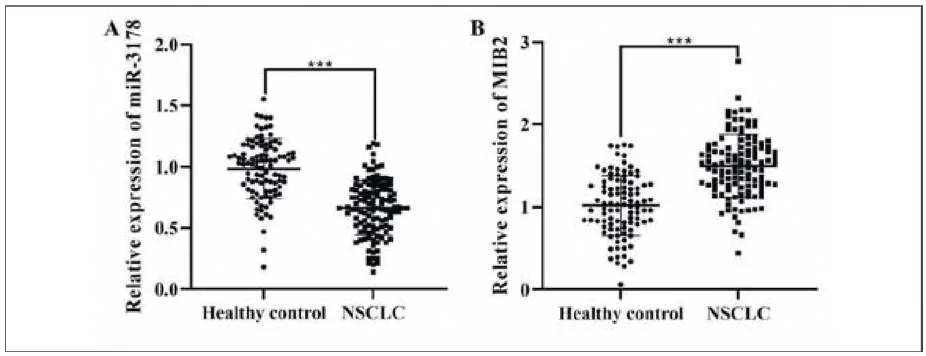
Expression levels of miR-3178 and MIB2 in the serum of NSCLC patients. A) The expression level of miR-3178 B) The expression level of MIB.

Compared to the control group, NSCLC patients exhibited significantly reduced miR-3178 expression levels (*P*<0.01, Figure 1A) and considerably elevated MIB2 expression levels (*P*<0.01, Figure 1B).

### Correlation of miR-3178 expression with clinical characteristics

Using the mean expression value of miR-3178 (0.66), 118 patients were divided into two groups: the high miR-3178 expression group and the low miR-3178 expression group. As shown in Table 2, the expression level of miR-3178 was significantly correlated with tumour-nodes-metastasis stage (TNM, *P* = 0.016) and lymph node metastasis (LNM, *P*=0.024) in NSCLC patients, while no significant correlation was observed with other characteristics, including age, gender, smoking history, drinking history, histology type, differentiation, and tumour size (*P*>0.05).

**Table 2:** The correlation between the expression level of miR-3178 and the clinical characteristics of patients. Note: TNM, tumour-nodes-metastasis; LNM, lymph node metastasis (negative, positive)

Variables	NSCLC patients<br>(n=118)	miR-3178 expression		*P*-value
		Low (n = 59)	High (n = 59)	
Age (years)				
<60	39	22	17	0.328
≥60	79	37	42	
Gender				
Male	71	37	34	0.573
Female	47	22	30	
Smoking history				
No	55	27	28	0.854
Yes	63	32	31	
Drinking history				
No	61	28	33	0.357
Yes	57	31	26	
Histology type				
Adenocarcinoma	79	38	41	0.557
Squamous cell carcinoma	39	21	18	
Differentiation				
High and moderate	101	49	52	0.432
Poor	17	10	7	
Tumour size				
<3	89	46	43	0.521
≥3	29	13	16	
TNM stage				
I	91	40	51	0.016
II-III	27	19	8	
LNM				
Negative	99	45	54	0.024
Positive	19	14	5	

### Prognostic analysis of NSCLC patients

**Figure 2:**
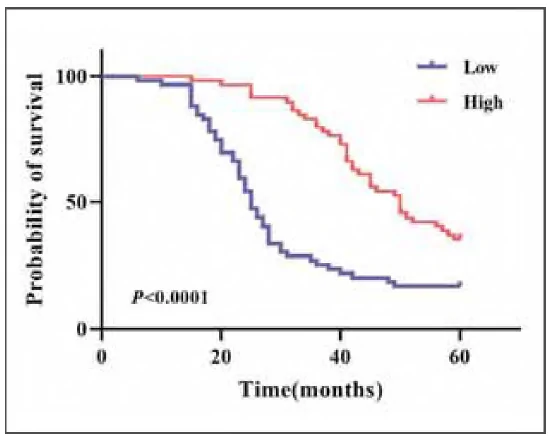
Kaplan-Meier Curves for patients with low or high miR-3178 expression.

A five-year follow-up and Kaplan-Meier survival analysis based on miR-3178 expression levels revealed that NSCLC patients with higher miR-3178 expression had significantly better 5-year overall survival rates compared to those with lower miR-3178 expression (*P*<0.0001, Figure 2). Univariate analysis showed that TNM stage (HR=1.985, 95% CI = 1.222-3.226, *P*=0.006), LNM (HR=1.863, 95% CI = 1.081-3.209, *P*=0.025), miR-3178 expression (HR=0.372, 95% CI = 0.242-0.573, *P*<0.001), and MIB2 expression (HR=1.92, 95% CI=1.252-2.947, *P*=0.003) were significantly associated with 5-year survival in NSCLC patients. Multivariate analysis confirmed TNM stage (HR=1.952, 95% CI = 1.183-3.22, *P*=0.009), miR-3178 expression (HR=0.418, 95% CI = 0.2670.654, *P*<0.001), and MIB2 expression (HR=1.576, 95% Ci = 1.017-2.443, *P*<0.042) as independent prognostic factors (Table 3).

**Table 3:** The correlation between the expression level of miR-3178 and the clinical characteristics of patients. Note: TNM, tumour-nodes-metastasis; LNM, lymph node metastasis

Variables	Univariate analysis			Multivariate analysis		
	HR	95% CI	P value	HR	95% CI	P value
Age	0.977	0.938-1.018	0.273			
Gender	0.843	0.436-0.843	0.436			
Smoking history	0.918	0.603-1.398	0.69			
Drinking history	0.995	0.653-1.517	0.982			
Histologic type	1.187	0.763-1.848	0.447			
Differentiation	0.928	0.504-1.707	0.81			
Size	1.121	0.696-1.806	0.639			
TNM stage	1.985	1.222-3.226	0.006	1.952	1.183-3.22	0.009
LNM	1.863	1.081-3.209	0.025	1.714	0.978-3.003	0.06
miR-3178 expression	0.372	0.242-0.573	<0.001	0.418	0.267-0.654	<0.001
MIB2 expression	1.92	1.252-2.947	0.003	1.576	1.017-2.443	0.042

### Target relationship between miR-3178 and MIB2

**Figure 3:**
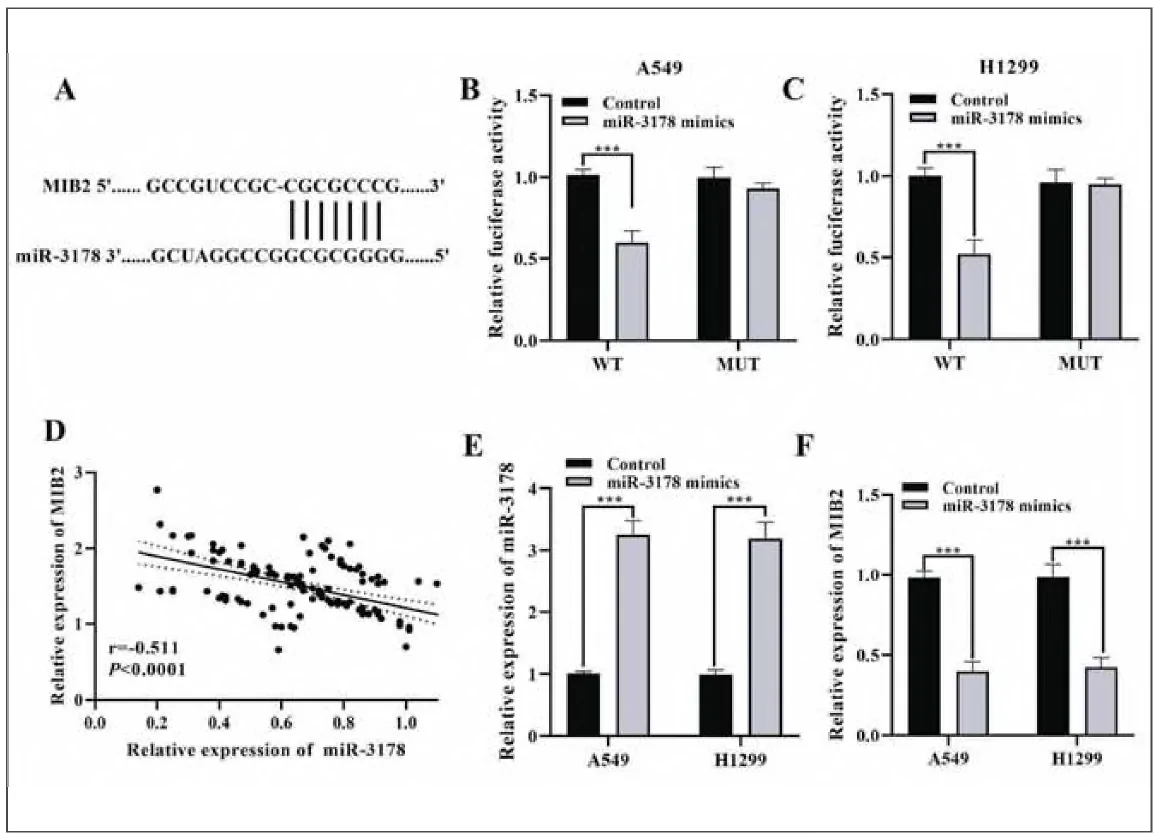
Luciferase reporter gene assay to verify the targeting relationship between miR-3178 and MIB2. A) Predicted targeting sites between miR-3178 and MIB2 B) Luciferase reporter gene assay in A549 cells C) Luciferase reporter gene assay in H1299 cells D) Correlation analysis between miR-3178 and MIB2 E) Expression levels of miR-3178 in cell lines after transfection F) Expression levels of MIB2 in cell lines after transfection.

Bioinformatic analyses using TargetScan (https://www.targetscan.org/vert_72/) predicted MIB2 as a target gene of miR-3178. miR-3178 could directly bind to the 3 UTR of MIB2 (Figure 3A). Overexpression of miR-3178 significantly reduced the luciferase activity of MIB2-WT in both A549 (Figure 3B) and H1299 (Figure 3C) cell lines (*P*<0.01), while no significant change was observed in MIB2-MUT (*P*>0.05). The expression levels of miR-3178 and MIB2 in the serum of NSCLC patients exhibit a significant negative correlation (*P*<0.01), with a correlation coefficient of -0.511. (*P*<0.01, Figure 3D). Overexpression of miR-3178 significantly increased miR-3178 expression (*P*<0.01, Figure 3E) and decreased MIB2 expression (*P*<0.01, Figure 3F) in both A549 and H1299 cell lines.

### Diagnostic value of miR-3178 and MIB2 alone and in combination

**Figure 4:**
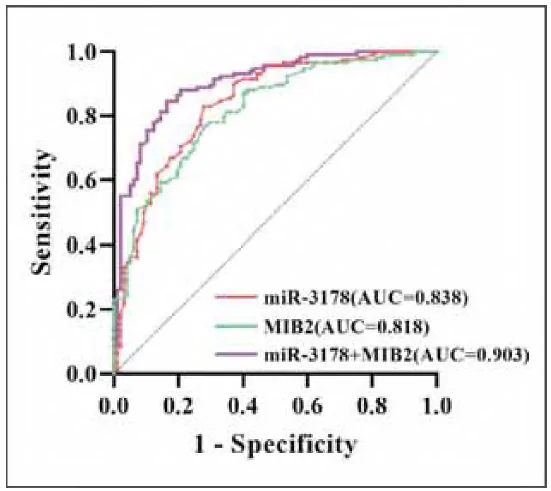
Diagnostic value of miR-3178 and MIB2 alone and in combination.

Figure 4 presents the diagnostic performance. The area under the curve (AUC) for miR-3178 alone was 0.838 (95% 0=0.784-0.892), with a sensitivity (positive true rate) of 83.1%, specificity of 72.2%, and false rate of 27.8%. For MIB2 alone, the AUC was 0.818 (95% CI=0.763-0.874), sensitivity of 76.3%, specificity of 72.2%, and false rate of 27.8%. When miR-3178 and MIB2 were combined, the AUC increased to 0.903 (95% CI=0.863-0.944), sensitivity of 84.8%, specificity of 83.5%, and false rate of 16.5%.

## Discussion

NSCLC, as the primary type of lung cancer, most patients have reached the middle or advanced stage at the time of treatment, thus missing the best opportunity for surgical intervention. Despite significant progress in treatment methods in recent years, the therapeutic effects and prognosis of patients remain poor [20][21]. Identifying miRNAs as biomarkers facilitates early diagnosis and targeted rapid treatment for NSCLC, thereby reducing patient mortality. miRNAs have emerged as biomarkers for various types of cancer. In the context of gastric cancer, the dysregulation of miR-19a and miR-199a has provided new diagnostic avenues for this malignancy [22].

Furthermore, miR-203 has been significantly downregulated and correlates strongly with multiple myeloma-related parameters [23]. A variety of miRNAs have been reported as potential biomarkers for NSCLC, with distinct characteristics shown by miR-630, miR-654-3p, and miR-199a-3p/5p [24][25][26]. Therefore, abnormal expression of miRNAs in the serum of NSCLC patients has been found in many studies to be useful for auxiliary diagnosis [27].

It has been reported that miR-3178 is a tumour-associated miRNA linked to various cancers, including lung cancer, prostate cancer, and hepatocellular carcinoma. In these cancers, the expression of miR-3178 is dysregulated [15][28][29]. Previous studies have shown that miR-3178 expression is significantly downregulated in tumour tissues of pancreatic cancer patients [30]. Downregulation of miR-3178 has been detected in patients with primary gastric cancer accompanied by positive LNM [31]. In gastric stromal tumours, miR-3178 expression is significantly down-regulated, leading to speculation that it may serve as a tumour suppressor gene for use in the diagnosis and prognosis of gastric stromal tumours [32]. Our results showed that the serum miR-3178 levels in NSCLC patients were significantly lower than those in healthy individuals, consistent with previous gene chip findings [14]. Studies have found that miR-3178 is downregulated in gastric cancer and significantly correlated with the patient's TNM stage and LNM [33]. This is similar to the findings of our study, low expression of miR-3178 was related to advanced clinical features of NSCLC, such as advanced TNM stage and higher incidence of LNM, which ultimately led to a poorer prognosis. Therefore, the expression of miR-3178 is closely related to the tumour progression and severity of NSCLC patients, and can potentially predict the progression of their condition.

Studies have demonstrated that tumour markers, such as ALK and PD-L1, serve as important prognostic indicators and overall survival assessment markers in spinal metastases of NSCLC [34]. However, while the use of standard tumour markers may improve the accuracy of identifying suitable surgical candidates, their effectiveness in predicting overall survival rates and diagnosing early-stage disease remains limited. Therefore, the exploration of new biomarkers is significant. Studies have found that the downregulation of miR-3178 is associated with poor prognosis, and miR-3178 and the patient's TNM stage are considered two independent prognostic indicators for gastric cancer [28][32]. Similar to the results of this study, which observed that NSCLC patients with low serum miR-3178 expression levels had lower survival rates and poor prognosis via Kaplan-Meier analysis. Numerous studies have also identified LNM and TNM staging as playing crucial roles in the diagnosis and prognosis of NSCLC [35][36]. Our study also confirms these findings. Through Cox regression analysis, we further discovered that the expression levels of miR-3178 and MIB2 remained independent prognostic predictors for NSCLC patients after adjusting for confounding factors.

In recent years, studies have found that E3 ligase is related to lung cancer cell proliferation and metastasis. MIB2, a member of the E3 ligase family, has attracted increasing attention in recent years. The expression trend of MIB2 varies across different types of tumours. For example, MIB2 has been reported to be up-regulated in leiomyomas [37], whereas in melanoma, it functions as a tumour suppressor [38][39][40]. MIB2 acts as a suppressor in lung cancer, where it has been found to inhibit the proliferation, migration, and invasion of NSCLC through ubiquitination of Notch1 [41]. MIB2 levels were significantly elevated, also aligning with prior research [42]. These findings suggest that miR-3178 and MIB2 have the potential to serve as diagnostic biomarkers for NSCLC. miR-3178 also has multiple targets during cancer progression. For instance, miR-3178 inhibits the proliferation, migration, and invasion of hepatocellular carcinoma by suppressing EGR3 expression while promoting its apoptosis [29]. Notch1 is one of the targets of miR-3178, as miR-3178 inhibits the progression of triple-negative breast cancer [28]. According to the prediction made by TargetScan, MIB2 is a target gene of miR-3178, and miR-3178 negatively regulates MIB2. Studies have shown that serum miR-145 is an excellent biomarker for distinguishing NSCLC patients from healthy control groups, with an AUC of 0.84, and optimal sensitivity and specificity were 92.75% and 61.43% [43]. Serum CRP demonstrates significant diagnostic value for NSCLC, with an AUC of 0.795 and diagnostic sensitivity and specificity of 81.8% and 66.8%, respectively [44]. Our study found that the AUC for miR-3178 alone in diagnosis is 0.838, with a sensitivity and specificity were 83.1% and 72.2% (44). This indicates that compared to established biomarkers, miR-3178 exhibits higher specificity for the diagnosis of NSCLC. Furthermore, the AUC for MIB2 alone was 0.818, with a sensitivity and specificity were 76.3% and 72.2%. When miR-3178 was used in combination with MIB2 for diagnosis, the AUC value reached 0.903, with sensitivity and specificity increasing to 84.8% and 83.5%, respectively. Therefore, the combined diagnostic approach of miR-3178 and MIB2 can be clinically adopted for the diagnosis of NSCLC.

However, this study still has certain limitations. For instance, we did not explore the roles of miR-3178 and MIB2 in the cellular biological functions of NSCLC, which could help reveal novel regulatory axes and molecular mechanisms, thereby providing new targets for the diagnosis and treatment of NSCLC. Although this study did not directly address this issue, we will pursue follow-up research in this promising direction.

In conclusion, we discovered that miR-3178 is significantly downregulated in NSCLC serum and cell lines. The downregulation of miR-3178 is correlated with the progression of NSCLC patients and indicates a poor prognosis. Therefore, miR-3178 has the potential to serve as a molecular marker for assessing the prognosis of NSCLC patients. Given that miR-3178 targets and regulates MIB2, the combined diagnosis of these two factors can enhance the diagnostic value for NSCLC. Monitoring the dynamic expression levels of both miR-3178 and MIB2 holds significant importance for analysing clinical pathological changes.

## Dodatak

### Acknowledgements

None.

### Authors' contributions

Deng Huang and Yingfang Ma contributed equally to this work.

### Conceptualisation

D.H., YM., H.Y, S.L., and L.Z.; Data curation: D.H., yM., H.Y, S.L., and L.Z.; Formal analysis: H.Y. and S.L.; Funding acquisition: S.L.; Investigation: H.Y. and S.L.; Methodology: D.H., Y.M., H.Y, S.L., and L.Z.; Project administration: S.L. and L.Z.; Resources: H.Y. and S.L.; Software: H.Y and S.L.; Supervision: S.L. and L.Z.; Validation: H.Y and S.L.; Visualisation: H.Y. and S.L.; Writing - original draft: H.Y; Writing - review and editing: D.H., Y.M., S.L., and L.Z.

### Funding

This research received no specific grant from funding agencies in the public, commercial, or not-for-profit sectors.

### Data availability

The datasets generated during and/or analysed during the current study are available from the corresponding author on reasonable request.

### Conflict of interest statement

All the authors declare that they have no conflict of interest in this work.
